# Frequency, persistence and relation of disease symptoms, psychosomatic comorbidity and daily life impairment after COVID-19: a cohort study in general practice

**DOI:** 10.1186/s12875-024-02551-w

**Published:** 2024-08-10

**Authors:** Alexander Hapfelmeier, Jan Donhauser, Clara Teusen, Stefanie Eck, Antonius Schneider

**Affiliations:** Institute of General Practice and Health Services Research, TUM School of Medicine and Health, Technical University of Munich, Munich, Germany

**Keywords:** SARS-CoV-2, General practice, Primary health care, Medically unexplained symptoms, Somatoform disorders

## Abstract

**Background:**

Long-lasting symptoms with a possible relation to psychosomatic comorbidity have been described following COVID-19. However, data is sparse in general practice. The trial’s objective was to investigate the time-dependent frequency of disease symptoms and relation to psychosomatic comorbidity and daily life impairment (DLI).

**Methods:**

Comparative cohort study of patients reporting a previous SARS-CoV-2 infection and uninfected controls in general practice. Participants were recruited in 14 general practices in the greater Munich area. Data collection was questionnaire based with a 12 months follow-up. Descriptive statistics, multivariable regression and bivariate correlations were used for analysis.

**Results:**

A total of *n* = 204 cases infected up to 42 months ago (*n* = 141 Omicron, *n* = 63 earlier variants), and *n* = 119 controls were included. Disease symptoms were substantially more prevalent in cases (55–79% vs. 43% within one year of infection). This difference also appeared in the multivariable analysis adjusting for socio-demographics and psychosomatic comorbidity with odds ratios (OR) of 4.15 (*p* < 0.001) and 3.51 (*p* = 0.054) for the cohorts with Omicron or earlier variants infection (vs. controls), respectively. It was persistent with earlier variants (OR 1.00 per month, *p* = 0.903), while a decreasing trend was observed for Omicron (OR 0.89 per month, *p* < 0.001). DLI was especially correlated with fatigue (*r* = 0.628).

**Conclusion:**

DLI, psychosomatic comorbidity and independently increased disease symptoms require holistic treatment of the patient in general practice according to the bio-psycho-social model. A key role in restoring the daily life capability may be attributed to the symptom fatigue.

**Supplementary Information:**

The online version contains supplementary material available at 10.1186/s12875-024-02551-w.

## Background

In the COVID-19 pandemic, the perspective has changed from an acute emergency situation to the long-term management of a better known and less severe disease, requiring better understanding and treatment of patients with long-term health problems [1, 2]. In this context, fatigue, dyspnoea, difficulty concentrating and many other long-lasting symptoms have been described, usually accompanied by daily life impairment (DLI) and reduced perceived quality of life [3]. Accordingly, long COVID syndrome (LCS) is defined by symptoms persisting over four weeks, while Post-COVID syndrome (PCS) describes new or persisting symptoms over three months after infection, impacting daily functioning [3].

Data on the prevalence of long-lasting symptoms are heterogeneous. Among the many reasons for such deviations are different recruitment of study populations and temporal effects [3, 4]. To name a few examples from the vast body of literature, one population-based study described persistent symptoms in 13.3% of SARS-CoV-2-infected patients 28 days after infection using health insurance registry data [5]. A cohort study reported a 4% prevalence twelve weeks after infection in school children [6]. In a meta-analysis of COVID symptoms including studies from diverse settings and populations [7] and in another cohort study of participants recruited through social media campaigns or personal invitation [8], 15% and 85% of long COVID cases remained affected at twelve months, respectively.

The nature of LCS or PCS has also been widely debated, with some studies suggesting functional responses to the pandemic rather than due to the disease itself [9, 10]. Others have shown that psychosomatic comorbidity is widely associated with long COVID [3, 11, 12]. Risk factors for the disease such as female sex, lack of vaccination, older age, obesity, infection with pre-Omicron variants or various pre-existing medical conditions are also discussed [1, 13–15]. However, the impact of psychosomatic comorbidity and patient selection on primary care remained unclear up to now.

The present study was conducted to investigate the prevalence and persistence of physical symptoms and DLI after SARS-CoV-2 infection with regard to possible risk factors and a possible dependence on psychosomatic comorbidity in general practice. See supplementary data S1 for a plain language summary.

## Methods

### Study design

This is a prospective comparative cohort study of cases reporting a previous SARS-CoV-2 infection and uninfected controls with a 12 months patient individual follow-up. The study was conducted in general practice. Data collection was questionnaire based. Each patient filled out a questionnaire upon enrolment, serving as the baseline assessment. Cases received two additional mailings with returns, 6 and 12 months after the baseline. Non-responders were reminded after two weeks to increase the response rate. First-patient-in was in March 2022 and last-patient-out was in November 2023.

### Recruitment and selection of study subjects

Patients were consecutively recruited between March and October 2022 in 14 general medical practices in Munich and the greater Munich area. Medical assistants or the participating medical student asked patients in the waiting rooms if they wanted to participate in the study and checked the inclusion criteria of age ≥ 18 years and sufficient German language skills. Eligible patients were fully informed about the study and their rights orally and in writing by their general practitioners and included upon giving written consent.

Participation of general practitioners and of a citizen advisory board is described in supplementary data S2.

### Measurements and outcomes

The questionnaires enquired about socio-demographics: age (years), height (cm), weight (kg) and gender (female/male/diverse). Standardized and validated questionnaires assessed anxiety, depression, and somatic symptom disorder (SSD), alongside symptom surveys for fatigue and dyspnoea to gauge symptom presence and severity, or somatic and psychosomatic comorbidity: Patient Health Questionnaire-15 (PHQ-15, [16]), Patient Health Questionnaire-4 (PHQ-4, [17]), Somatic Symptom Disorder - B Criteria Scale (SSD-12, [18]), Fatigue Assessment Scale (FAS, [19]), and modified dyspnoea scale of the Medical Research Council (mMRC, [20, 21]) (see supplementary data S3 and the original publications of these questionnaires for details).

Presence of well-described and common symptoms was also queried (each yes/no) [3, 22]. These were fatigue, shortness of breath, chest pain, memory problems, concentration problems, sleep disturbance, palpitations, dizziness, depression, anxiety, tinnitus, loss of appetite, weight loss, cough, headache, change in sense of smell, change in sense of taste, skin rashes and muscle pain. Cases were additionally asked about the timing of their SARS-CoV-2 infection (month and year) and other factors that were considered to be related to the symptom burden of the COVID-19 disease or to result from it, that is vaccination status at the time of infection (yes/no), additional infections between surveys (yes/no), and daily life impairment (yes/no). No repeated symptom surveys were carried out for the controls, as it was likely that they would also become infected in between the survey times. More importantly, without reference to an infection date in the control group, we would not have been able to obtain any meaningful information about a time-dependent course. Vaccination status was also not investigated in the controls, as the research question relating to vaccination was its association with the specific burden of the COVID-19 disease, which was not present in the controls.

Data management is described in supplementary data S4.

### Statistical analysis

The distribution of data is presented by descriptive statistics. Trends over time are described for six-month periods since month of infection in accordance with the intervals between the survey times. The cohort of cases was further divided into sub-cohorts most likely infected with the Omicron variant or earlier variants, as these showed markedly different effects. The division was based on 31 December 2021 on which the Omicron variant almost completely and immediately replaced the earlier variants in Germany [23]. Group differences were tested using t-tests, analysis of variance (ANOVA) and chi-squared tests.

Multivariable binary logistic regression models were used to estimate the time-dependent frequency of reported symptoms in relation to the SARS-CoV-2 infection status (yes ‘Omicron’/yes ‘earlier variant’/no), the time measured in months since infection (corresponding to a value of zero in the controls) and their interaction. Other predictors of interest were the socio-demographics and the reported psychosomatic comorbidity. The inclusion of height and weight was preferred to the inclusion of body mass index (BMI) in order to improve the goodness of fit of the models [24]. The models were also extended to analyse the relation of the burden of the COVID-19 disease to vaccination prior to infection and further infections that occurred between the survey times in the cohorts of cases. All models were pre-specified. The occurrence of any symptoms or the frequent symptoms of fatigue, dyspnoea, impaired concentration and memory problems were considered to be of primary interest. Effect estimates are expressed as odds ratios (OR) or translated into marginal event probabilities. Respective hypothesis tests and 95% confidence intervals were adjusted for repeated measures using a clustered covariance matrix for the models’ parameter estimates [25, 26].

Descriptive network analyses were constructed to illustrate bivariate correlations and partial correlations [27]. To increase clarity and interpretability, only correlations of moderate size (|r|≥0.3) are displayed.

Statistical analysis was performed using R 4.3.0 (The R Foundation for Statistical Computing, Vienna, Austria). All tests were two sided with exploratory 5% significance levels. Sample size considerations are given in supplementary data S5.

## Results

### Study population

**Fig. 1:**
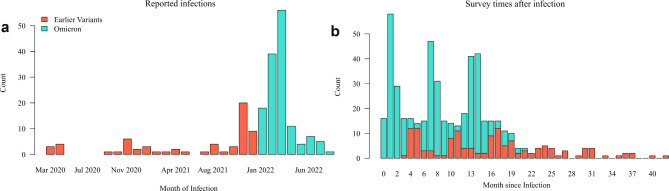
(**A**) Frequency distribution of reported infections between March 2020 and August 2022. Colour indicates the division of cases into a cohort with Omicron infection and a cohort with infection by earlier variants using 31 December 2021 as the cut-off date. (**B**) Frequency distribution of the survey times as months since the reported infection, covering a total of 42 months

Included were *n* = 204 cases (*n* = 141 Omicron; *n* = 63 earlier variants), and *n* = 119 controls. Cases reported infections between March 2020 and August 2022 (Fig. 1A). The reported infection times aligned with the incidence of COVID-19 in Germany during this period [28]. Recruitment and follow-up resulted in survey times reaching from 0 to 21 months and 3 to 42 months since infection, respectively (Fig. 1B). There were 145 (71.1%) and 146 (71.6%) cases responding at the two follow-up survey times and 167 (81.9%) responding at least once.

The control cohort was more than 10 years older (*p* < 0.001), the Omicron cohort included about 8–9% more women (*p* = 0.308) and distribution of weight and height were comparable (Table 1). Among the cases, the Omicron cohort more frequently reported a previous vaccination (*p* < 0.001), which may be due to the increasing availability of the vaccine over the course of the pandemic. No diverse gender was reported; thus, the variable was converted to sex (female/male). All variables presented here were included in the multivariable analysis.

**Table 1:** Socio-demographic data of the cohorts at baseline. Indices give the number of missing values

	Controls (*n* = 119)	Omicron (*n* = 141)	Earlier variants (*n* = 63)	*p*-value
Age (years)	59.1 ± 14.9^1^	48.6 ± 15.1	48.2 ± 16.7^1^	< 0.001
Sex (female)	71 (59.7%)	95 (67.4%)	36 (58.1%)^1^	0.308
Height	171 ± 9	170 ± 9	171 ± 9	0.706
Weight	74.5 ± 19.4^3^	76.4 ± 21.1^1^	77.6 ± 18.1^2^	0.581
Previous vaccination	–	135 (96.4%)^1^	24 (38.7%)^1^	< 0.001

### Descriptive statistics

The frequency of any symptoms or the symptoms of fatigue, dyspnoea, impaired concentration and memory problems, and DLI, are shown in Table 2. Direct cohort comparison was feasible for up to two years after infection. Results show that disease symptoms were substantially more frequent in cases compared to controls. Over time, this frequency decreased in the Omicron cohort, but remained high in the cohort with infections with earlier virus variants. High frequency and differing trends could also be observed for DLI. Statistics for all investigated symptoms and the entire observation period are available in supplementary data S6. These results reflect the effects described above and show the persistence of symptoms in the earlier virus variant cohort over a long period (supplementary data S6).

Cases also exhibited higher frequency of mMRC (moderate/severe) and higher sum scores of SSD-12, FAS, PHQ-15 and PHQ-4, assessing perceived dyspnoea, fatigue, somatisation disorders, anxiety and depression (Table 2). No obvious time trends could be recognised in this analysis. Statistics on the whole observation period are available in supplementary data S6.

**Table 2:** Descriptive statistics on disease symptoms defined to be of primary interest, DLI, psychosomatic comorbidity, mMRC and FAS, per cohort and summarised for four half-years since infection, in which a direct comparison is possible. Statistics for further symptoms and up to the eighth half-year are presented in supplementary data S6. Indices give the number of missing values

			Time since infection in half-years (*N* of Omicron/Earlier variants)			
	Controls (*N* = 119)	Cases	1 (124/25)	2 (108/27)	3 (113/33)	4 (12/23)
Disease symptoms						
Any symptom	50 (43%)	Omicron	90 (74%)	59 (55%)	58 (52%)^1^	5 (42%)
		Earlier	19 (79%)	21 (78%)	20 (61%)	15 (68%)
Fatigue	19 (16%)	Omicron	59 (48%)^1^	43 (40%)	41 (37%)^2^	3 (25%)
		Earlier	12 (48%)	13 (50%)^1^	11 (33%)	13 (57%)
Concentration/memory*	14 (12%)^1^	Omicron	38 (31%)^3^	26 (24%)	22 (20%)^1^	3 (25%)
		Earlier	11 (48%)^2^	11 (41%)	11 (33%)	9 (41%)
Impaired concentration	11 (9%)^1^	Omicron	38 (31%)^3^	22 (20%)	21 (19%)^1^	3 (25%)
		Earlier	10 (43%)^2^	10 (37%)	10 (30%)	9 (39%)
Memory problems	9 (8%)^1^	Omicron	22 (18%)^3^	18 (17%)	17 (15%)^1^	2 (17%)
		Earlier	10 (43%)^2^	8 (30%)	10 (30%)	6 (29%)^2^
Dyspnoea	12 (10%)^1^	Omicron	31 (26%)^5^	13 (12%)	13 (12%)^2^	1 (8%)
		Earlier	9 (36%)	5 (19%)	9 (27%)	7 (32%)^1^
DLI	NA	Omicron	51 (41%)	32 (30%)^3^	28 (25%)^1^	3 (25%)
		Earlier	11 (44%)	7 (26%)	12 (39%)^2^	11 (48%)
Psychosomatic comorbidity						
SSD-12	7.8 ± 8.3^7^	Omicron	11.2 ± 10.1	11.5 ± 10.9^2^	11.2 ± 10.8	15.2 ± 11.4
		Earlier	13.5 ± 10.2	10.9 ± 9.9	12.7 ± 10.7^1^	13.9 ± 9.6
PHQ-15	5.2 ± 4.4^7^	Omicron	7.0 ± 5.2^3^	6.1 ± 5.2^11^	6.0 ± 5.2^4^	6.0 ± 4.6^2^
		Earlier	7.3 ± 6.7^1^	6.9 ± 4.9^1^	6.5 ± 5.4^3^	9.0 ± 7.5^1^
PHQ-4	1.8 ± 2.1^1^	Omicron	2.6 ± 2.7	2.5 ± 2.9^2^	2.2 ± 2.6^1^	2.6 ± 2.5
		Earlier	2.8 ± 2.5	2.1 ± 2.6	2.4 ± 2.5	3.2 ± 2.6^1^
mMRC (moderate/severe)	4 (4%)^7^	Omicron	18 (16%)^12^	12 (13%)^16^	8 (8%)^15^	1 (10%)^2^
		Earlier	2 (8%)	4 (17%)^3^	3 (11%)^6^	4 (20%)^3^
FAS	18.2 ± 5.7^12^	Omicron	22.9 ± 8.2	21.4 ± 7.8^1^	21.3 ± 7.4	22.4 ± 7.2
		Earlier	24.6 ± 10.6	23.3 ± 8.2	23.6 ± 8.3^1^	24.0 ± 8.7^1^

### Multivariable regression models

**Fig. 2:**
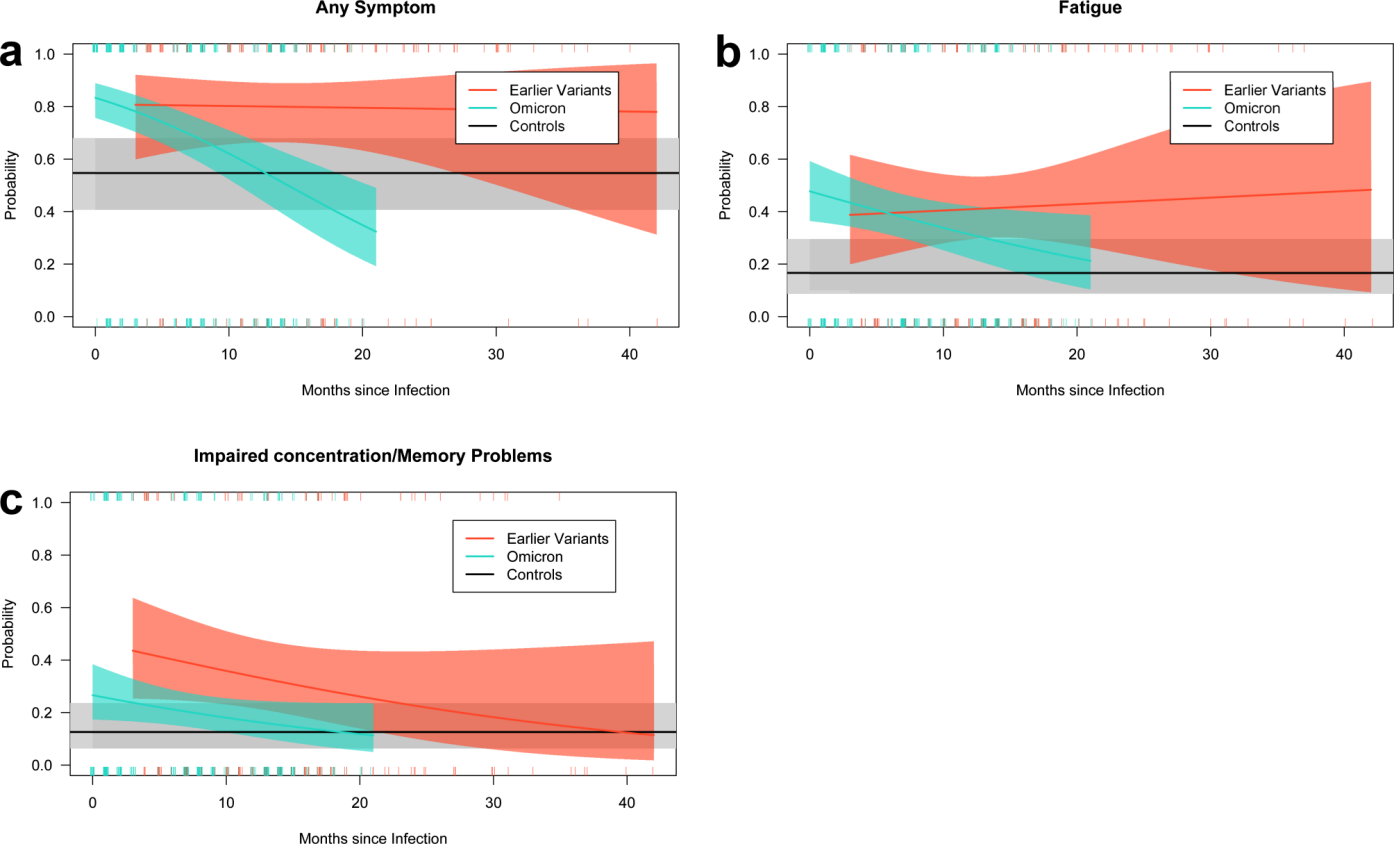
Probability (solid line) and pointwise 95% confidence intervals (background colouring) of the outcomes ‘any symptom’ (**A**), fatigue (**B**) and impaired concentration or memory problems (**C**) for each cohort in dependence of time measured as months since infection. Ticks indicate the frequency distribution of outcomes (top = ‘outcome reported’, bottom = ‘outcome not reported’). Estimates are derived from the multivariable logistic regression models reported in Table 3

Probabilities of the occurrence of any symptoms, fatigue, impaired concentration and memory problems, estimated by multivariable regression models, are shown in Fig. 2. The models are presented in Table 3. Despite being frequently observed, dyspnoea could not be considered in this analysis as the number of records was not sufficient to fit the large multivariable model. Case cohorts with Omicron or earlier virus variant infection were more likely to report any disease symptom in the initial months after infection compared to controls, with estimated OR of 4.15 (*p* < 0.001) and 3.51 (*p* = 0.054) in the month of infection. A decreasing trend was observed in the Omicron cohort (OR 0.89 per month, *p* < 0.001), which approached the control cohort over time, while the earlier virus variant cohort showed a persistent effect (OR 1.00 per month, *p* = 0.903). Psychosomatic comorbidity was associated with an increased risk, with the PHQ-15 reaching statistical significance (OR 1.21, *p* < 0.001). Additional analyses of case cohorts regarding prior vaccination or further infections between the survey times showed numerical increases in risk (OR 1.18, *p* = 0.800 and OR 2.63, *p* = 0.035; models not shown).

**Table 3:** Multivariable binary logistic regression models including a presentation of odds ratios with 95% confidence intervals (95%-CI). *„Concentration/memory“ = ‚Impaired concentration‘ or ‚Memory problems‘. ^a^Time since infection with Omicron variant. ^b^Time since infection with earlier variants

		Regression coefficients			Odds ratios		
Outcome	Predictor variables	Estimate	Std. error	p-value	Estimate	95%-CI Lower	95%-CI Upper
Any Symptom	Intercept	2.568	3.760	0.495			
	Earlier variants	1.256	0.653	0.054	3.512	0.537	22.948
	Omicron	1.424	0.360	< 0.001	4.154	1.474	11.704
	PHQ-15	0.193	0.045	< 0.001	1.213	1.065	1.381
	SSD-12	0.037	0.019	0.060	1.037	0.981	1.097
	FAS	0.045	0.027	0.101	1.046	0.967	1.132
	mMRC (moderate/severe)	-0.118	0.638	0.854	0.889	0.142	5.569
	PHQ-4	0.058	0.079	0.465	1.060	0.844	1.331
	Age (years)	0.003	0.009	0.712	1.003	0.977	1.031
	Height (cm)	-0.031	0.022	0.160	0.970	0.910	1.033
	Sex (male)	0.181	0.419	0.666	1.198	0.360	3.993
	Weight (kg)	-0.003	0.009	0.748	0.997	0.973	1.022
	Time (month) x Omicron^a^	-0.112	0.021	< 0.001	0.894	0.841	0.950
	Time (month) x Earlier^b^	-0.004	0.035	0.903	0.996	0.900	1.102
Fatigue	Intercept	0.749	3.747	0.842			
	Earlier variants	1.125	0.698	0.107	3.079	0.415	22.840
	Omicron	1.521	0.452	0.001	4.578	1.252	16.734
	PHQ-15	0.111	0.036	0.002	1.117	1.007	1.239
	SSD-12	0.023	0.021	0.278	1.023	0.963	1.087
	FAS	0.085	0.022	< 0.001	1.088	1.020	1.161
	mMRC (moderate/severe)	0.583	0.467	0.212	1.792	0.469	6.841
	PHQ-4	0.042	0.073	0.570	1.042	0.845	1.286
	Age (years)	0.003	0.010	0.804	1.003	0.973	1.033
	Height (cm)	-0.030	0.021	0.165	0.971	0.913	1.032
	Sex (male)	-0.385	0.436	0.378	0.680	0.194	2.381
	Weight (kg)	-0.005	0.007	0.529	0.995	0.974	1.017
	Time (month) x Omicron^a^	-0.058	0.026	0.026	0.944	0.876	1.017
	Time (month) x Earlier^b^	0.010	0.039	0.796	1.010	0.904	1.129
Concentration/memory*	Intercept	-4.908	4.022	0.222			
	Earlier variants	1.816	0.618	0.003	6.145	1.043	36.216
	Omicron	0.923	0.484	0.057	2.516	0.626	10.106
	PHQ-15	0.078	0.039	0.044	1.081	0.967	1.207
	SSD-12	0.048	0.020	0.017	1.049	0.990	1.112
	FAS	0.108	0.025	< 0.001	1.115	1.037	1.198
	mMRC (moderate/severe)	-0.503	0.387	0.194	0.605	0.199	1.839
	PHQ-4	-0.053	0.064	0.404	0.948	0.789	1.139
	Age (years)	0.009	0.010	0.382	1.009	0.980	1.040
	Height (cm)	0.001	0.023	0.971	1.001	0.937	1.069
	Sex (male)	-0.315	0.493	0.524	0.730	0.177	3.010
	Weight (kg)	-0.011	0.009	0.221	0.990	0.965	1.014
	Time (month) x Omicron^a^	-0.050	0.028	0.077	0.951	0.878	1.031
	Time (month) x Earlier^b^	-0.046	0.031	0.142	0.955	0.873	1.045

### Network analysis

**Fig. 3:**
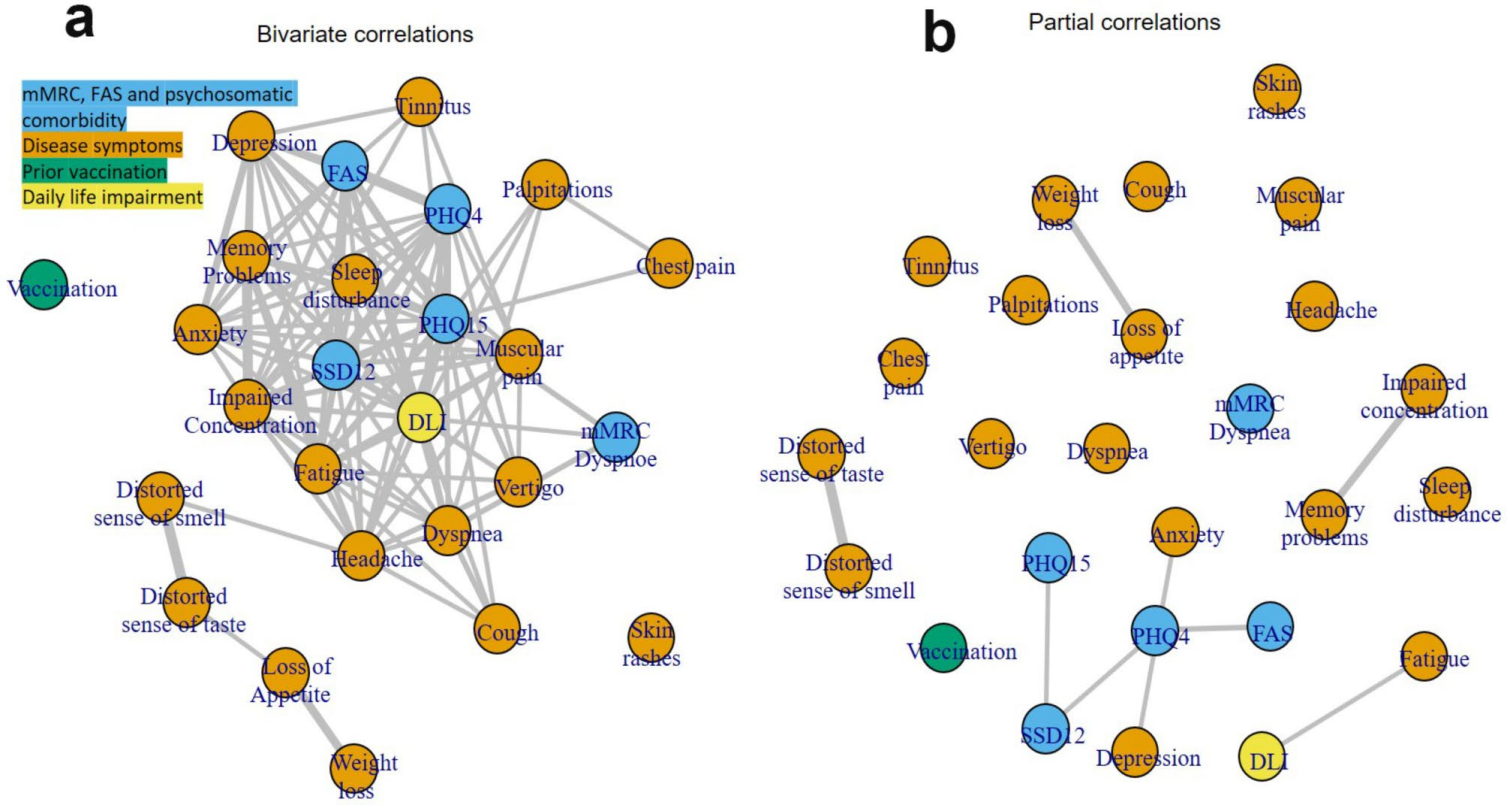
(**A**) Bivariate and (**B**) partial correlations with a size of at least |r|≥0.3 between DLI (yellow), disease symptoms (orange), mMRC, FAS and psychosomatic comorbidity (blue) and prior vaccination (green). Increasing size of correlation is indicated by closer neighbour-ship and width of edges. Numeric values of correlations are given in supplementary data S7

Results of the network analysis in case cohorts revealed numerous moderate to strong correlations of DLI with disease symptoms and psychosomatic comorbidity (Fig. 3A; see supplementary data S7 for all numerical values). Strongest correlations were observed with fatigue (*r* = 0.628), SSD-12 (*r* = 0.583), dyspnoea (*r* = 0.539) and PHQ-15 (*r* = 0.515). The only significant direct or partial correlation, meaning that it could not be explained by indirect correlations via other variables, existed with fatigue (*r* = 0.338, Fig. 3B; see also supplementary data S7 for all numerical values).

## Discussion

### Main findings

We found symptom persistence in more than half of the patients after SARS-CoV-2 infection, recruited from primary care. As a concomitant effect, these patients reported considerably more physical comorbidity than controls, independently of an equally substantially higher level of psychological comorbidity. DLI was identified as another prevalent problem showing strongest correlations with fatigue, dyspnoea, SSD-12 and PHQ-15. These effects were particularly persistent with earlier virus variants.

### Strengths and limitations

Inclusion of controls enables symptom frequency comparison. To our knowledge, there is no comparative evaluation in primary care up to now. However, a nocebo effect cannot be ruled out since a ‘placebo infection’ cannot be simulated in the controls. Speculation could arise that symptoms may have developed solely due to knowledge of infection. However, this seems unlikely given the large differences between cohorts, symptom persistence and the typical symptom patterns of fatigue, dyspnoea and impaired concentration. Furthermore, a selection bias cannot be ruled out in this observational study, as the controls were on average about 10 years older than the cases. However, it is known that older age is a risk factor for the disease [1, 14]. Therefore, the even better results in the older controls suggest that the derived differences between the groups may even be conservative estimates. In addition, the group comparisons in the multivariable analyses were adjusted for age and other sociodemographic data to reduce potential bias. The questionnaire-based survey may have led to recall bias, especially regarding infections and vaccination, and information bias, for example due to question misunderstanding. These sources of bias may have led to both an underestimation and an overestimation of effects, for example in relation to the potentially protective or harmful effects of vaccination and several previous infections, or in relation to the frequency of symptoms. Even with 81.9% of the cases responding in the follow-up, attrition bias remains possible, which can potentially lead to an accumulation or reduction of more severely affected persons and consequently to over- or underestimation of symptom frequency. However, we did not observe a decrease in responses between the second and third survey, with many participants responding to one or the other survey. The division of cases into sub-cohorts may have led to allocation bias and an underestimation of respective group differences. However, the Omicron variant almost completely and immediately displaced earlier variants in Germany around 31 December 2021 [23], and only 9 (4%) and 18 (9%) cases reported an infection in December 2021 and January 2022, respectively. The complex multivariable analyses could not be carried out for each symptom due to the study’s limited sample size. Corresponding models were intentionally not created to prevent bias. The severity of disease could not be taken into account, for example by use of the National Institutes of Health (NIH) clinical spectrum of SARS-CoV-2 infection ranging from no symptoms to critical illness, as clinical assessment or imaging was not available in the present study [29]. The control questionnaire excluded COVID-19-specific questions about vaccination status and DLI. In retrospect, this information could have been used for additional analyses that go beyond the research question of the relation to the burden of the COVID-19 disease. No repeated surveys were conducted on controls, as the trend over time was linked to the infection event. Possible effects due to the calendar timeline could therefore not be controlled.

### Relation to existing literature

Population-based surveys indicate DLI prevalence of 5–28% in patients 12 to 18 months after SARS-CoV-2 infection [30]. DLI frequency is significantly higher in our primary care study, exceeding 50%. This aligns with healthcare system selection mechanisms [31], as more patients naturally consult their general practitioner for persistent comorbidity. Patient suffering is evident when compared to controls. Cases had considerably more symptoms, greater impairment of everyday life and markedly higher psychological comorbidity, although the controls were on average 10 years older. This clear difference is remarkable given that there are studies in which the so-called long COVID or post-COVID-19 syndrome symptoms are attributed to placebo or, better, nocebo effects [32–34].

The comparison with controls could contribute to better understanding of LCS or PCS. Of course, psychological effects play a major role in the development of symptoms after infections. On the one hand, increased psychological comorbidity is a predictor for the development of post-infectious symptoms, as has also been shown by routine data analysis for other infectious diseases [35, 36]. However, these analyses indicate psychosomatic comorbidity as an independent predictor, showing no significant interaction with the infectious event. In addition, expectations play a significant role, as negative symptom expectations and processing can create tension that almost invites symptoms to develop [32], which is accompanied by excessive preoccupation with the disease in terms of somatic symptom disorder. However, a pure nocebo effect seems unlikely, as the controls had fewer symptoms and a significantly lower disease burden. Rather, it seems obvious that symptoms, especially fatigue, significantly contribute to the patients’ impaired mental state as indicated by our network analysis and results of our earlier population-based study [11].

### Implications for research and practice

Notably, earlier variants exhibit persistent fatigue, dyspnoea, and concentration impairment, whereas the Omicron variant shows quicker symptom decline. This could contribute to a more differentiated assessment and prognosis of the time course of individual patients, although further research on symptom development over time and risk factors is required. These findingsmay be attributed to vaccination effects [37], with broader population coverage during the Omicron variant emergence, or increasing infestation [38]. However, the extent of mental comorbidity remains rather constant over time. As vaccination and infestation continue to offer partial protection at best, managing PCS remains a challenge in primary care, particularly due to the lack of specific treatments. Impaired mental health might at least partly be explained by persistent unexplained and subjectively disturbing symptoms in the new clinical picture of PCS, which are challenging to treat [11]. Therefore, in addition to treating the symptoms, the high level of psychological distress should also be considered, in line with the bio-psycho-social model, corresponding to general practitioner heuristics [39]. Numerous studies, some of which could also be suitable for GP practices, are currently being conducted to find out which psychological interventions can best support patients [40]. Beyond that, development of patient guidelines and manuals could also be useful to enable patient empowerment [41, 42]. Finally, as far as fatigue is concerned, staged activation and pacing appear to be the most promising [3]. Further studies would need to investigate how this can best be achieved in practice, for example using activating training apps [43]. In addition to stepwise activation and pacing [3], appropriate psychological interventions could be useful in this regard.

## Conclusion

The multitude of complex relations and independent effects of disease symptoms, psychosomatic comorbidity and DLI emphasizes that patients of general practitioners need holistic treatment. The symptom of fatigue may have a key role in establishing suitability for everyday life. Therefore, stepwise activation and pacing [3] and additional appropriate psychological interventions may be indicated in these difficult-to-treat patients.

### Electronic supplementary material

Below is the link to the electronic supplementary material.

Supplementary Material 1

## Data Availability

The written consent obtained from patients does not authorise the disclosure of study data to third parties, for which there is no legal basis. However, the extent to which anonymised data, e.g. aggregated data, may be shared can be reviewed upon reasoned request to the corresponding author.
