# Supplementary material for: Frequency, persistence and relation of disease symptoms, psychosomatic comorbidity and daily life impairment after COVID-19: a cohort study in general practice
Source: BMC Prim Care. 2024 Aug 10;25:295. doi: 10.1186/s12875-024-02551-w (PMC11316996; doi:10.1186/s12875-024-02551-w)
Supplement: Supplementary file 1 — Supplementary Material 1 [file 12875_2024_2551_MOESM1_ESM.pdf]

S1: Plain Language Summary

In this study, we asked 204 patients who reported a previous coronavirus infection to complete a five-page questionnaire about their illness and their remaining physical and psychological symptoms. We repeated the survey by mail after six and twelve months. In addition, we asked 119 patients who had not yet experienced a coronavirus infection to complete an almost identical questionnaire. All patients were recruited from 14 general practices in the greater Munich area. By comparing the two groups, it was possible to investigate whether disease symptoms were elevated beyond a normal level. Due to the repeated answers to the questionnaires and the different lengths of time since the infection, the study was able to cover a long period of time. Relations to age, sex, virus variants, additional infections and previous vaccination were also explored.

We found that patients with an infection reported physical and psychological symptoms more frequently than patients who were not yet infected. We also found that this frequency remained high for a long time in patients infected with earlier variants of the virus. In contrast, the frequency of health comorbidity in patients infected with the omicron variant approached that of patients without infection over time. It is important to note that these are only general trends and that individual patients may have shorter or even longer lasting symptoms. We found no evidence of a strong influence of sex, age, previous vaccination or another coronavirus infection between the survey dates on these results. However, this does not mean that these effects can generally be ruled out.

Our analyses also showed that psychological stress appears to be one but not the only possible cause of the symptoms. This contradicts the assumption that long-lasting symptoms after COVID-19 are exclusively due to psychological causes. On the contrary, it could even be that psychological comorbidity is partly a consequence of stressful physical symptoms.

We also analysed the relation of symptoms with limitations in daily life and found that the strongest relation was with fatigue, which was also the most commonly reported symptom in the study.

Based on our findings, we recommend that both psychological and physical long-lasting symptoms of COVID-19 should be taken seriously and treated appropriately in general practice. Special consideration should be given to the significant impact of fatigue on limitations in daily life.

S2: Participation

The study design was presented to the general practitioners of the 14 participating practices in a face-to-face meeting by the principal investigator before the study protocol was finalised. Important feedback, for example on the design of a control group and on organisational procedures, could thus be incorporated into the study planning.

After conduct of the study, seven members of the patient and citizen advisory board (23) affiliated with the Institute of General Practice and Health Services Research of the Technical University of Munich were involved in providing feedback on the study results and their presentation. The advisory board consists of patients and members of the general public. It acts as an advisory body to evaluate research projects at the Institute of General Practice and Health Services Research from the perspective of the target audiences of research in general medicine. During a face-to-face meeting, members of the advisory board developed new research questions that would be of interest to them in the context of the study, for example about the effects of age and gender, and prioritized these together with previously developed research questions mentioned in the study protocol. In addition, members of the advisory board were asked about their preferred methods of presenting the study results and recommendations for practice in the research article. The patient and citizen advisory board's suggestions were included if they did not conflict with the study protocol or as additional analyses and presentations.

S3: Standardised and validated questionnaires

- The Patient Health Questionnaire-15 (PHQ-15) is part of the PHQ-D health questionnaire and assesses the presence and severity of frequent somatic symptoms, according to the SSD A criterion, within the last 4 weeks using 15 items. The total score of the PHQ-15 ranges from 0 to 30, and cut-off scores of 5, 10, 15 can be used to define the severity of somatic symptoms as low, medium and high (15).
- The Patient Health Questionnaire-4 (PHQ-4) is a two-dimensional instrument for assessing depression and anxiety. It combines the PHQ-2, which consists of two core diagnostic criteria of the DSM-IV for depressive disorders, and the GAD-2, which consists of the two core criteria of the GAD-7 for generalised anxiety disorder, into a composite four-item scale. The PHQ-4 total score ranges from 0 to 12. Total scores of 6 and 9 were recommend as "yellow and red flags" for the presence of a depressive or anxiety disorder in clinical practice (16).
- The Somatic Symptom Disorder - B Criteria Scale (SSD-12) consists of 12 items to measure the B-criteria of somatoform disorders according to DSM-V (psychological symptom burden in the context of somatoform disorders). Each of the three psychological sub-criteria of SSD (cognitive, affective and behavioural aspects) is measured with four items. The total score of the SSD-12 ranges from 0 to 48, and a cut-off score of 23 has been suggested (17).
- The Fatigue Assessment Scale (FAS) is a 10-item questionnaire for assessing the severity of fatigue. Five questions each relate to physical and mental fatigue. The answers are mapped on a 5-point scale (from "1= never" to "5= always"). The total score on the FAS can therefore be 10-50 points. Scores of 10-21 indicate no fatigue, scores of 22-50 indicate significant fatigue, with scores ≥ 35 indicating extreme fatigue (18).
- The modified dyspnoea scale of the Medical Research Council (mMRC) divides the degree of breathlessness during daily activities into four categories according to self-assessment: "0 = Not troubled with breathlessness except with strenuous exercise", "1 = Troubled by shortness of breath when hurrying on the level or walking up a slight hill", "2 = Walks slower than people of the same age on the level because of breathlessness or has to stop for breath when walking at own pace on the level", "3 = Stops for breath after walking about 100 yards or after a few minutes on the level" and "4 = Too breathless to leave the house or breathless when dressing or undressing". A grade of 2, 3 or 4 on the dyspnoea scale corresponds to moderately to severely debilitating COPD (19-21).

S4: Data management

The study was approved by the local ethics committee of the Technical University of Munich before conduct. The data collected was entered into a database that was stored in the secure systems of the University Hospital of the Technical University of Munich and was only accessible to authorised study personnel. Data of the controls was stored in anonymised form, as no personalised data was required to contact them again after the baseline survey. De-identifying data of the cases were stored in a separate file from the study data. In rare cases of missing, incorrect or ambiguous information, a consensus decision was reached between two raters as to whether it would be possible to impute or correct this information. For example, it was possible to fill in missing information about symptoms if a patient wrote as a comment that they had no symptoms, or it was possible to derive a conservative result from ambiguous information, for example if someone had ticked "severe" and "very severe" in relation to dyspnoea. In such a case, the perceived dyspnoea was obviously also "very severe". A missing value was assigned whenever the unambiguousness could not be clarified in the consensus of both raters.

S5: Sample size

A sample size of n=200 cases and n=100 controls was defined for the study to enable consistent estimation of multivariable regression models with at least three parameters to describe the time-dependent frequency of disease symptoms in the cases compared to the controls (24). At the time of study planning in autumn 2021, approximately 10-20% of previously infected persons in Germany suffered from long COVID and should therefore show corresponding disease symptoms, as inquired in the present study. On average, this would correspond to the minimum necessary number of 30 cases with symptoms required for the estimation of three parameters, whereby controls with symptoms would further add to this number. The feasibility of the recruitment was given, as 20 patients were to be recruited in 10 participating practices, which in turn could be recruited in the Bavarian Practice Based Research Network (BayFoNet) (25). BayFoNet currently comprises more than 100 general practices in Bavaria, Germany, and is funded by the Federal Ministry of Education and Research for the structural support of research in general practice (01GK1903A-D).

Table S6: Descriptive statistics of symptoms and psychosomatic comorbidity

	Controls (N=119)	Cases	Time since infection in half-years (N of Omicron/Earlier variants) 1 (124/25)	2 (108/27)	3 (113/33)	4 (12/23)	5 (0/14)	6 (0/10)	7 (0/5)	8 (0/1)
DLI	NA	Omicron	51 (41%)	32 (30%)	28 (25%)	3 (25%)				
		Earlier	11 (44%)	7 (26%)	12 (39%)	11 (48%)	7 (50%)	3 (30%)	2 (40%)	0 (0%)
Any symptom	50 (43%)	Omicron	90 (74%)	59 (55%)	58 (52%)	5 (42%)				
		Earlier	19 (79%)	21 (78%)	20 (61%)	15 (68%)	11 (79%)	9 (90%)	3 (60%)	0 (0%)
Fatigue	19 (16%)	Omicron	59 (48%)	43 (40%)	41 (37%)	3 (25%)				
		Earlier	12 (48%)	13 (50%)	11 (33%)	13 (57%)	9 (64%)	6 (60%)	2 (40%)	0 (0%)
Concentration/memory*	14 (12%)	Omicron	38 (31%)	26 (24%)	22 (20%)	3 (25%)				
		Earlier	11 (48%)	11 (41%)	11 (33%)	9 (41%)	6 (43%)	4 (40%)	0 (0%)	0 (0%)
Impaired concentration	11 (9%)	Omicron	38 (31%)	22 (20%)	21 (19%)	3 (25%)				
		Earlier	10 (43%)	10 (37%)	10 (30%)	9 (39%)	6 (43%)	4 (40%)	0 (0%)	0 (0%)
Memory Problems	9 (8%)	Omicron	22 (18%)	18 (17%)	17 (15%)	2 (17%)				
		Earlier	10 (43%)	8 (30%)	10 (30%)	6 (29%)	6 (43%)	3 (30%)	0 (0%)	0 (0%)
Headache	14 (12%)	Omicron	46 (38%)	23 (21%)	18 (16%)	1 (8%)				
		Earlier	9 (36%)	6 (22%)	8 (24%)	7 (32%)	6 (43%)	2 (22%)	0 (0%)	0 (0%)
Cough	7 (6%)	Omicron	49 (40%)	19 (18%)	20 (18%)	1 (8%)				
		Earlier	6 (26%)	7 (26%)	7 (21%)	6 (26%)	2 (14%)	3 (30%)	1 (20%)	0 (0%)
Sleep disturbance	17 (14%)	Omicron	30 (25%)	19 (18%)	18 (16%)	1 (8%)				
		Earlier	8 (32%)	7 (26%)	4 (12%)	4 (18%)	4 (29%)	4 (40%)	1 (20%)	0 (0%)
Dyspnoea	12 (10%)	Omicron	31 (26%)	13 (12%)	13 (12%)	1 (8%)				
		Earlier	9 (36%)	5 (19%)	9 (27%)	7 (32%)	3 (21%)	3 (30%)	2 (40%)	0 (0%)
Muscular pain	10 (8%)	Omicron	29 (24%)	16 (15%)	14 (12%)	2 (17%)				
		Earlier	7 (28%)	5 (19%)	4 (12%)	5 (22%)	6 (43%)	3 (30%)	1 (20%)	0 (0%)
Vertigo	11 (9%)	Omicron	21 (18%)	16 (15%)	13 (12%)	2 (17%)				
		Earlier	5 (23%)	6 (22%)	3 (9%)	5 (22%)	4 (29%)	3 (30%)	1 (20%)	0 (0%)
Palpitations	9 (8%)	Omicron	21 (18%)	8 (8%)	11 (10%)	0 (0%)				
		Earlier	4 (17%)	6 (22%)	4 (12%)	2 (9%)	0 (0%)	1 (11%)	0 (0%)	0 (0%)
Distorted sense of smell	3 (3%)	Omicron	10 (8%)	8 (7%)	7 (6%)	1 (8%)				
		Earlier	7 (30%)	4 (15%)	4 (12%)	4 (18%)	3 (21%)	1 (10%)	0 (0%)	0 (0%)
Anxiety	10 (8%)	Omicron	7 (6%)	9 (8%)	8 (7%)	0 (0%)				
		Earlier	7 (29%)	4 (15%)	5 (15%)	3 (13%)	3 (21%)	3 (30%)	0 (0%)	0 (0%)
Depression	11 (9%)	Omicron	9 (7%)	10 (10%)	10 (9%)	0 (0%)				
		Earlier	6 (26%)	2 (7%)	4 (12%)	4 (17%)	1 (7%)	2 (25%)	0 (0%)	0 (0%)
Chest pain	2 (2%)	Omicron	16 (13%)	8 (7%)	6 (5%)	0 (0%)				
		Earlier	4 (17%)	3 (11%)	5 (15%)	1 (4%)	2 (14%)	0 (0%)	1 (20%)	0 (0%)
Distorted sense of taste	5 (4%)	Omicron	9 (8%)	5 (5%)	5 (5%)	1 (8%)				
		Earlier	8 (35%)	3 (11%)	5 (15%)	5 (22%)	3 (21%)	1 (10%)	0 (0%)	0 (0%)
Tinnitus	9 (8%)	Omicron	6 (5%)	7 (7%)	5 (4%)	0 (0%)				
		Earlier	3 (14%)	1 (4%)	0 (0%)	5 (22%)	1 (8%)	3 (30%)	0 (0%)	0 (0%)
Loss of appetite	3 (3%)	Omicron	10 (8%)	4 (4%)	0 (0%)	0 (0%)				
		Earlier	4 (17%)	4 (15%)	1 (3%)	2 (9%)	1 (7%)	1 (10%)	0 (0%)	0 (0%)
Weight loss	3 (3%)	Omicron	6 (5%)	4 (4%)	1 (1%)	0 (0%)				
		Earlier	3 (13%)	4 (15%)	1 (3%)	2 (9%)	1 (7%)	1 (10%)	0 (0%)	0 (0%)
Skin rashes	4 (3%)	Omicron	6 (5%)	3 (3%)	4 (4%)	0 (0%)				
		Earlier	3 (13%)	2 (7%)	3 (9%)	0 (0%)	1 (7%)	1 (10%)	0 (0%)	0 (0%)
mMRC (moderate/ severe)	4 (4%)	Omicron	18 (16%)	12 (13%)	8 (8%)	1 (10%)				
		Earlier	2 (8%)	4 (17%)	3 (11%)	4 (20%)	2 (17%)	2 (29%)	0 (0%)	0 (0%)
FAS	18.2±5.7	Omicron	22.9±8.2	21.4±7.8	21.3±7.4	22.4±7.2				
		Earlier	24.6±10.6	23.3±8.2	23.6±8.3	24.0±8.7	23.3±10.7	24.2±8.7	24.0±6.7	14.0
SSD-12	7.8±8.3	Omicron	11.2±10.1	11.5±10.9	11.2±10.8	15.2±11.4				
		Earlier	13.5±10.2	10.9±9.9	12.7±10.7	13.9±9.6	15.0±14.3	12.7±14.6	12.0±11.0	0.0
PHQ-15	5.2±4.4	Omicron	7.0 ±5.2	6.1±5.2	6.0±5.2	6.0±4.6				
		Earlier	7.3±6.7	6.9±4.9	6.5±5.4	9.0±7.5	8.1±7.8	8.4±7.3	11.3±9.2	3.0
PHQ-4	1.8±2.1	Omicron	2.6±2.7	2.5±2.9	2.2±2.6	2.6±2.5				
		Earlier	2.8±2.5	2.1±2.6	2.4±2.5	3.2±2.6	3.1±3.1	2.5±3.1	2.0±1.4	0.0

Values are „number (%)“ or Mean ± standard deviation per half-year since infection. Symptoms are sorted by overall frequency in decreasing order. For a full reporting of the numbers of missing values see supplementary data S8. *„Concentration/memory“ = ‚Impaired concentration‘ or ‚Memory problems‘.

Table S7: Bivariate correlations

	Fatigue	Dyspnoe	Chest pa	Memory	Impaired	Sleep dis	Palpitatic	Vertigo	Depressi	Anxiety	Tinnitus	Loss of a	Weight lo	Cough	Headach	Distortec	Distortec	Skin rash	Muscula	PHQ-15	SSD-12	FAS	mMRC	PHQ-4	Prior vac	DLI
Fatigue	1	0.389	0.220	0.520	0.526	0.444	0.297	0.371	0.320	0.279	0.288	0.242	0.155	0.352	0.448	0.240	0.215	0.104	0.372	0.526	0.453	0.477	0.261	0.415	-0.122	0.628
Dyspnoe	-0.011	1	0.286	0.265	0.330	0.265	0.344	0.246	0.195	0.317	0.135	0.162	0.096	0.423	0.370	0.259	0.200	0.162	0.291	0.455	0.388	0.361	0.417	0.287	-0.148	0.539
Chest pain	0.016	0.091	1	0.102	0.111	0.170	0.324	0.133	0.114	0.159	0.127	0.024	0.071	0.170	0.201	-0.054	0.005	0.169	0.223	0.379	0.204	0.201	0.182	0.185	-0.034	0.270
MemoryProblems	0.172	-0.069	-0.031	1	0.753	0.322	0.267	0.286	0.488	0.411	0.325	0.213	0.154	0.176	0.401	0.222	0.236	0.056	0.372	0.385	0.442	0.448	0.177	0.380	-0.191	0.443
Impaired concentration	0.083	0.100	-0.043	0.581	1	0.406	0.278	0.296	0.403	0.358	0.315	0.261	0.130	0.244	0.427	0.233	0.234	0.107	0.409	0.437	0.485	0.484	0.178	0.393	-0.162	0.437
Sleep disturbance	0.137	-0.046	-0.027	-0.132	0.124	1	0.295	0.297	0.360	0.331	0.274	0.249	0.184	0.265	0.375	0.230	0.203	0.192	0.438	0.416	0.422	0.324	0.152	0.391	-0.099	0.435
Palpitations	0.009	0.094	0.227	0.057	0.017	0.140	1	0.209	0.237	0.284	0.180	0.059	0.011	0.273	0.230	0.181	0.203	0.073	0.240	0.352	0.269	0.293	0.231	0.201	0.001	0.321
Vertigo	0.094	-0.051	-0.047	0.010	-0.007	-0.015	0.046	1	0.230	0.296	0.281	0.291	0.172	0.212	0.413	0.288	0.202	0.144	0.359	0.449	0.333	0.221	0.283	0.314	-0.101	0.340
Depression	-0.063	-0.061	-0.029	0.218	-0.018	0.111	0.021	-0.025	1	0.536	0.333	0.109	0.105	0.134	0.262	0.186	0.127	0.066	0.306	0.371	0.394	0.414	0.067	0.427	-0.120	0.380
Anxiety	-0.088	0.152	0.033	0.071	-0.046	0.003	0.123	0.085	0.332	1	0.252	0.262	0.259	0.176	0.285	0.260	0.241	0.058	0.354	0.380	0.479	0.400	0.137	0.454	-0.199	0.356
Tinnitus	0.012	-0.127	-0.015	0.037	0.057	0.041	0.013	0.059	0.152	0.017	1	0.137	0.153	0.163	0.268	0.146	0.132	0.070	0.352	0.375	0.246	0.240	0.244	0.207	-0.153	0.263
Loss of appetite	0.020	-0.033	-0.068	-0.066	0.159	0.027	-0.024	0.125	-0.059	0.042	-0.050	1	0.636	0.260	0.254	0.284	0.307	0.162	0.244	0.172	0.177	0.089	0.223	0.206	-0.098	0.249
Weight loss	0.013	-0.044	0.090	0.081	-0.111	0.045	-0.107	-0.056	-0.012	0.153	0.061	0.582	1	0.214	0.174	0.251	0.237	0.133	0.206	0.139	0.106	0.036	0.130	0.061	-0.075	0.142
Cough	0.115	0.215	-0.061	-0.083	0.014	-0.003	0.114	-0.060	0.006	-0.037	0.022	0.053	0.087	1	0.390	0.181	0.205	0.245	0.261	0.359	0.289	0.208	0.285	0.229	0.020	0.350
Headache	0.049	0.089	0.055	0.102	0.066	0.080	-0.062	0.159	-0.027	-0.006	0.027	0.034	-0.024	0.190	1	0.300	0.269	0.097	0.399	0.476	0.375	0.281	0.200	0.311	-0.075	0.416
Distorted sense of smell	0.013	0.105	-0.164	-0.075	0.005	0.018	0.012	0.146	0.096	-0.008	-0.008	-0.050	0.105	-0.082	0.077	1	0.771	0.086	0.235	0.245	0.263	0.160	0.171	0.136	-0.209	0.289
Distorted sense of taste	-0.018	-0.077	0.028	0.082	-0.012	-0.015	0.091	-0.106	-0.100	0.047	-0.016	0.134	-0.049	0.089	0.008	0.723	1	0.098	0.245	0.238	0.213	0.151	0.122	0.114	-0.248	0.228
Skin rashes	-0.060	0.023	0.117	-0.027	0.026	0.088	-0.051	0.051	0.023	-0.084	-0.027	0.033	0.031	0.156	-0.081	-0.006	0.028	1	0.204	0.183	0.168	0.092	0.170	0.147	-0.023	0.152
Muscular pain	-0.007	0.019	0.056	0.037	0.072	0.175	-0.020	0.079	-0.004	0.087	0.137	0.014	0.026	-0.003	0.073	-0.029	0.065	0.092	1	0.470	0.402	0.294	0.211	0.312	-0.102	0.369
PHQ-15	0.127	0.074	0.230	-0.101	-0.007	0.005	0.040	0.195	0.076	-0.091	0.139	-0.091	0.057	0.048	0.150	-0.053	0.079	-0.011	0.121	1	0.693	0.624	0.411	0.577	-0.206	0.515
SSD-12	-0.082	-0.019	-0.071	0.041	0.099	0.061	-0.040	-0.042	-0.088	0.169	-0.071	-0.058	-0.005	0.032	-0.028	0.106	-0.053	0.041	0.041	0.347	1	0.664	0.296	0.700	-0.166	0.538
FAS	0.094	0.021	-0.054	-0.001	0.154	-0.094	0.106	-0.145	0.053	-0.001	-0.019	-0.119	0.042	-0.050	-0.076	-0.030	0.010	-0.031	-0.047	0.212	0.119	1	0.274	0.705	-0.248	0.506
mMRC	-0.062	0.209	-0.019	0.066	-0.080	-0.067	0.067	0.089	-0.125	-0.067	0.149	0.134	-0.023	0.062	-0.090	0.052	-0.071	0.062	-0.016	0.169	0.000	0.039	1	0.231	-0.101	0.376
PHQ-4	0.011	-0.059	0.025	0.012	-0.088	0.108	-0.106	0.092	0.099	0.126	-0.043	0.190	-0.154	0.025	0.019	-0.052	-0.023	0.043	-0.035	0.039	0.322	0.431	-0.013	1	-0.132	0.457
Prior vaccination	0.001	-0.103	-0.017	-0.078	0.029	-0.041	0.147	-0.009	0.034	-0.093	-0.082	-0.038	0.030	0.105	0.054	-0.016	-0.134	-0.005	0.041	-0.071	0.031	-0.173	-0.012	0.077	1	-0.095
DLI	0.338	0.270	0.101	0.065	-0.078	0.105	-0.016	0.017	0.118	-0.029	0.019	0.080	-0.050	0.002	0.030	0.068	-0.015	-0.002	0.007	-0.088	0.147	0.114	0.128	-0.013	0.092	1

Bivariate correlations (above diagonal) and partial correlations (below diagonal) of disease symptoms, DLI, psychosomatic comorbidity and prior vaccination. Correlations of size $|r| \geq 0.3$ are highlighted in green.

Table S8: Number of missing values.

	Controls	Time since infection in half-years (N of Omicron/Earlier variants)							
		1	2	3	4	5	6	7	8
	(N=119)	(124/25)	(108/27)	(113/33)	(12/23)	(0/14)	(0/10)	(0/5)	(0/1)
DLI	NA	0/0	3/0	1/2	0/0	-/0	-/0	-/0	-/0
Any symptom	2	2/1	1/0	2/0	0/1	-/0	-/0	-/0	-/0
Fatigue	0	1/0	0/1	2/0	0/0	-/0	-/0	-/0	-/0
Concentration/memory*	1	3/2	0/0	1/0	0/0	-/0	-/0	-/0	-/0
Impaired concentration	1	3/2	0/0	1/0	0/0	-/0	-/0	-/0	-/0
Memory Problems	1	3/2	0/0	1/0	0/2	-/0	-/0	-/0	-/0
Headache	3	2/0	1/0	2/0	0/1	-/0	-/1	-/0	-/0
Cough	2	3/2	1/0	3/0	0/0	-/0	-/0	-/0	-/0
Sleep disturbance	1	4/0	1/0	1/0	0/1	-/0	-/0	-/0	-/0
Dyspnoea	1	5/0	0/0	2/0	0/1	-/0	-/0	-/0	-/0
Muscular pain	1	4/0	2/0	1/0	0/0	-/0	-/0	-/0	-/0
Vertigo	0	4/3	0/0	1/0	0/0	-/0	-/0	-/0	-/0
Palpitations	1	5/1	2/0	1/0	0/0	-/0	-/1	-/0	-/0
Distorted sense of smell	2	5/2	1/0	1/0	0/1	-/0	-/0	-/0	-/0
Anxiety	0	5/1	1/0	1/0	0/0	-/0	-/0	-/0	-/0
Depression	0	3/2	5/0	2/0	0/0	-/0	-/2	-/0	-/0
Chest pain	0	3/1	1/0	2/0	0/0	-/0	-/0	-/0	-/0
Distorted sense of taste	0	4/2	0/0	2/0	0/0	-/0	-/0	-/0	-/0
Tinnitus	0	4/3	1/1	1/0	0/0	-/1	-/0	-/0	-/0
Loss of appetite	0	5/2	1/0	2/0	0/0	-/0	-/0	-/0	-/0
Weight loss	0	5/2	2/0	1/1	0/0	-/0	-/0	-/0	-/0
Skin rashes	0	6/2	0/0	1/0	0/0	-/0	-/0	-/0	-/0
mMRC (moderate/ severe)	7	12/0	16/3	15/6	2/3	-/2	-/3	-/1	-/1
FAS	12	0/0	1/0	0/1	0/1	-/0	-/0	-/0	-/0
SSD-12	7	0/0	2/0	0/1	0/0	-/1	-/1	-/0	-/0
PHQ-15	7	3/1	11/1	4/3	2/1	-/2	-/2	-/2	-/0
PHQ-4	1	0/0	2/0	1/0	0/1	-/1	-/0	-/0	-/0

Complementary table to Table S6 showing the number of missing values. *„Concentration/memory“ = ‚Impaired concentration‘ or ‚Memory problems‘.
